# The Influence of the Extremely Low Frequency Electromagnetic Field on Clear Cell Renal Carcinoma

**DOI:** 10.3390/ijms22031342

**Published:** 2021-01-29

**Authors:** Aleksandra Cios, Martyna Ciepielak, Wanda Stankiewicz, Łukasz Szymański

**Affiliations:** 1 Department of Microwave Safety, Military Institute of Hygiene and Epidemiology, 01-163 Warsaw, Poland; aleksandra.cios@wihe.pl (A.C.); martyna.ciepielak@wihe.pl (M.C.); wanda.stankiewicz@gmail.com (W.S.); 2 Department of Molecular Biology, Institute of Genetics and Animal Biotechnology, Polish Academy of Science, 05-552 Magdalenka, Poland

**Keywords:** electromagnetic field, EMF, ccRCC, kidney, renal carcinoma, cancer

## Abstract

The development of new technologies and industry is conducive to the increase in the number and variety of electromagnetic field (EMF) sources in our environment. The main sources of EMF are high-voltage lines, household appliances, audio/video devices, mobile phones, radio stations, and radar devices. In the growing use of electronic devices, scientists are increasingly interested in the effects of EMF on human health. Even though many studies on the effects of EMF have already been carried out, none of them has shown a significant effect on mammals, including humans. Moreover, it is not entirely clear how EMF influences cell behavior. The International Agency for Research on Cancer on 31 May 2011, classified PEM as a possible carcinogenic factor. This study aimed to investigate the effect of the electromagnetic field on morphological and functional changes in clear cell renal carcinoma. The research was carried out on in vitro cultures of four cell lines: HEK293, 786-O 769-P, and Caki1. The results of the research showed that the EMF of low frequency had a slight effect on the viability of cells. EMF, which induced cell arrest in the G1 phase, increased the number of early apoptotic cells and decreased the number of viable cells in the 786-O line. EMF did not affect the proliferation and viability of HEK293 cells. Extreme low-frequency EMF (ELF-EMF) also showed an inhibitory effect on the migration and metastatic properties of clear cell kidney cancer cells. Moreover, shortly after the end of ELF-EMF exposure, significant increases in ROS levels were observed in all tested cell lines. As part of the work, it was shown that low-frequency EMF shows an inhibitory effect on the proliferation of primary cancer cells, diminishing their migratory, invasive, and metastatic abilities. It also increases the apoptosis of cancer cells and the amount of reactive oxygen species. Based on the results of our research, we want to point up that the effect of ELF-EMF depends on a specific metabolic state or at a specific stage in the cell cycle of the cells under study.

## 1. Introduction

The extremely low-frequency electromagnetic field (ELF-EMF) is a field with a frequency of 0–300 Hz generated mainly by power lines. The magnetic induction, when exposed to very low-frequency electromagnetic fields, is typically below 0.1 μT [1]. No studies have shown that the electromagnetic field directly affects the human organism, exerting a chronic effect on our health. The influence of EMF on the human body, especially with regard to cancer, has become one of the major research topics. In the Monarchy IARC assessment of 2001, ELF-EMF and static electric and magnetic fields were classified as Group 3, which means that there is not enough scientific evidence to classify the agent as carcinogenic. Few studies related to these evaluations have been carried out since 2001, and none of them warrants a re-evaluation, as recently reflected in a meeting of the European Commission expert panel [1].

Research shows that if cancer cells are exposed to an electromagnetic field, their apoptosis will begin without causing cell necrosis [2]. Moreover, it is unclear how ELF-EMF would influence cell behavior. A likely and interesting hypothesis is that they can interfere with the chemical reactions involved in the production of reactive oxygen species (ROS). ROS are not only harmful to cellular structures but are also essential for cell signaling. High levels of ROS can lead to impairment of physiological functions of cells by damaging DNA, proteins, phospholipids, and other macromolecules. Inversely, at low levels, ROS may alter intracellular redox states that activate redox-sensitive pathways, including the MAPK and PI3K pathways, and consequently also the mTOR pathway [3,4].

The molecular mechanisms explaining the biological effects of EMFs have been studied in a variety of cell lines within the strict range of electromagnetic field performance parameters. Exposure to EMF has been proposed to reduce cell proliferation by increasing cell apoptosis due to an EMF-dependent increase in reactive oxygen species, rapid Ca^2+^ influx, or activation of specific signaling pathways [5]. Some researchers suggest non-specific processes such as heat generation, free radical formation, or promoting the creation of DNA damage. However, the energy normally associated with ELF-EMF is not sufficient to cause such changes in chemical bonds. However, neither of these mechanisms explains the differences in EMF response between cancer and non-malignant cells [6].

Despite this, the therapeutic properties of EMF have been scientifically confirmed many times in the treatment of lower back pain, multiple sclerosis, injuries of joints and tendons, and nerve regeneration in Parkinson’s and Alzheimer’s disease [7,8,9].

So far, no studies have been conducted to check the impact of EMF exposure on clear cell renal cell carcinoma cell lines. Therefore, in this study, we wanted to assess potential changes in the morphology and functioning of the cell lines we selected.

## 2. Results

### 2.1. 4.5 mT, 50 Hz ELF-EMF Did Not Affect Cell Morphology and Slightly Decreased Cell Viability

Exposure to 4.5 mT, 50 Hz ELF-EMF did not affect cell morphology and contraction. In order to investigate the effect of low-frequency EMF exposure on the cell growth rate, cell viability was determined 72 h after the end of EMF exposure. An in vitro TOX8 test employing resazurin was used for this study. Based on the obtained results, with the use of the formula presented in the materials and methods section, the percentage of cell viability was calculated. We observed that the viability of tumor cells exposed to ELF-EMF is lower compared to the control group (the greatest decrease in viability was noticed in 786-O cell line where it dropped by nearly 30%, in 769-P cell line by 19%, and in Caki1 by 15%), while the viability of the HEK293 line is higher by 27% compared to the control.

### 2.2. 4.5 mT, 50 Hz ELF-EMF Induced G0/G1 Arrest and Apoptosis in RCC Cells

**Figure 1:**
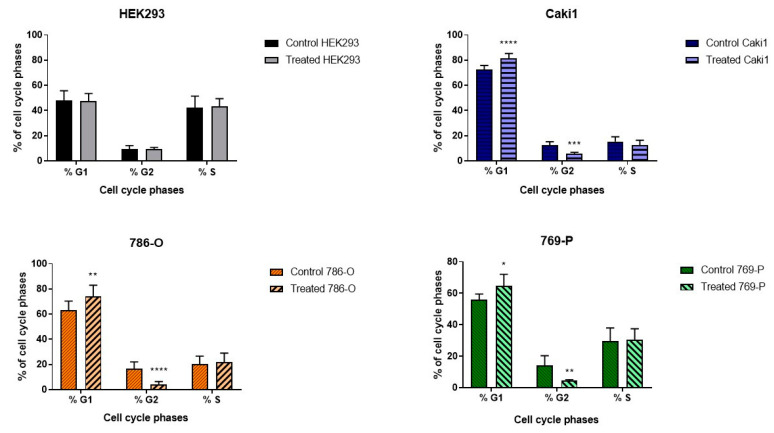
Results of cytometric analysis of cell cycle phases under the influence of 4.5 mT, 50 Hz extreme low-frequency electromagnetic field (ELF-EMF). Data presented as mean ± standard deviation: *—*p* < 0.05, **—*p* < 0.005, ***—*p* < 0.001, **** *p* < 0.0001.

Cytometric analysis performed on the fifth day after the end of ELF-EMF exposure showed that, in the tumor cell lines exposed to low-frequency EMF, the G1 cell population was larger, and the G2 population was smaller compared to cells of the sham group. The cytometric analysis allowed for the percentage determination of the number of cells in the G1, G2, and S phase (Figure 1). The obtained data indicate that low-frequency electromagnetic radiation inhibits the proliferation of tumor cells, which leads to the death of ccRCC cells.

What is more, 5 days after the exposure, cells were subjected to cytometric analysis to determine the type of cell death using double staining of cells with propidium iodide (PI) and annexin V.

**Figure 2:**
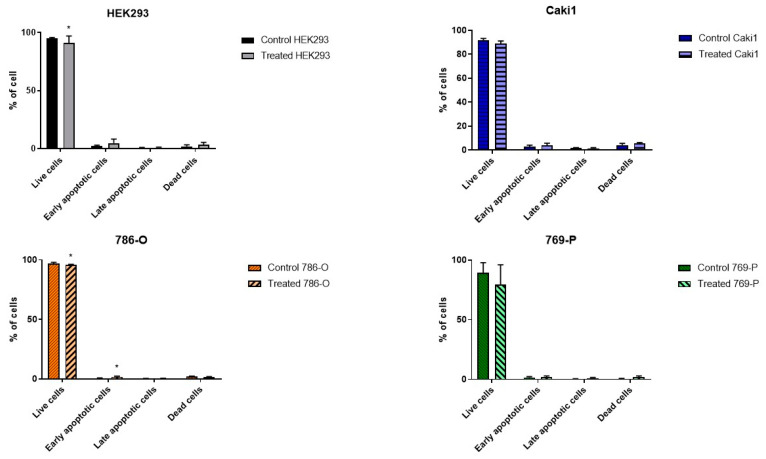
Results of the cytometric analysis of cell apoptosis under the influence of 4.5 mT, 50 Hz ELF-EMF. Data presented as mean ± standard deviation: *—*p* < 0.05.

4.5 m T, 50 Hz ELF-EMF, which induced cell arrest in the G1 phase, increased the number of early apoptotic cells and decrease the number of viable cells in line 786-O (Figure 2). Although in the remaining ccRCC lines, the changes in the cell population were similar, no statistically significant differences were observed.

The data obtained from evaluating the density plots in 9 replicates of each exposed and non-exposed line are represented by bar plots. No statistically significant differences were noticed in the HEK293 line, which would indicate that low-frequency EMF does not affect the proliferation and viability of healthy kidney cells.

### 2.3. 4.5 mT, 50 Hz ELF-EMF Induce Higher Production of ROS

**Figure 3:**
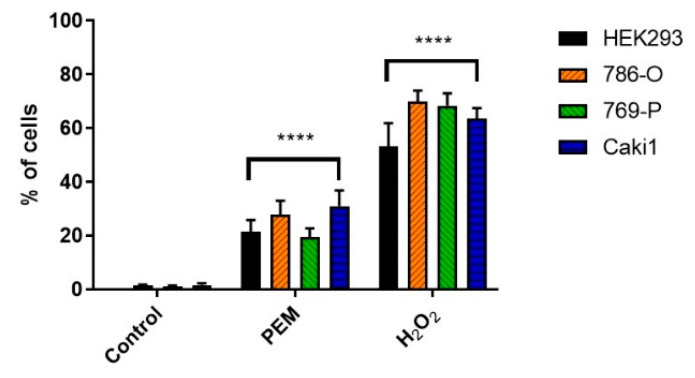
The results of the cytometric analysis of the% cells that produced reactive oxygen species under the influence of 4.5 mT, 50 Hz ELF-EMF. Data presented as mean ± standard deviation: **** *p* < 0.0001.

To assess the contribution of ROS to the biological effects of ELF-EMF, we measured intracellular ROS in ELF-EMF treated HEK293, 786-O, 769-P, and Caki1 cells compared to the sham group incubated for 2 h with H_2_O_2_. The cells positive for reactive oxygen species production were analyzed with the CellROX Green Reagent fluorogenic probes (Thermo Fisher Scientific, Warsaw, Poland). We observed that cells exposed to electromagnetic radiation generate much more ROS compared to the control group (Figure 3). This is also applied to the HEK293 line.

### 2.4. 4.5 mT, 50 Hz ELF-EMF Inhibits Migratory and Invasive Abilities of ccRCC Cell Lines

To determine the differences in metastatic capacity, the Delta Optical IB-100 microscope was used to count the cells that migrated towards the chemoattractant, which was the medium with 10% FBS.

**Figure 4:**
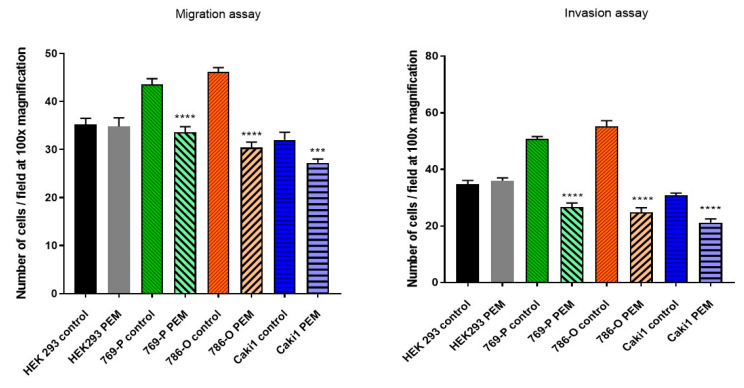
Number of cells migrating towards chemoattractant (average of 5 fields at 100× total magnification). (***—*p* < 0.0005; ****—*p* < 0.00005).

Results present on the graph bar (Figure 4) show that low-frequency EMF significantly inhibits the migratory and invasive abilities of tumor cells, without changing these properties in healthy cells.

### 2.5. 4.5 mT, 50 Hz ELF-EMF Inhibits Sphere/Spheroid Formation

**Figure 5:**
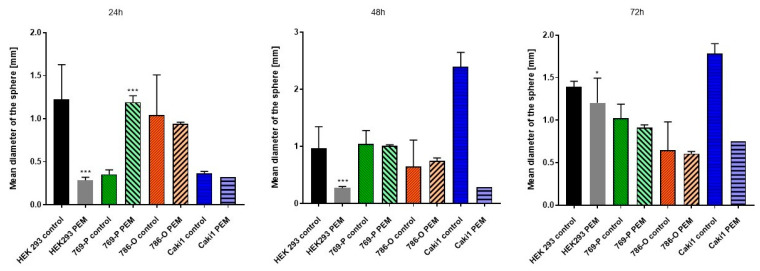
The analysis of the diameter of the spheroid was carried out in 24, 48, and 72 h, the mean values of the diameters of the spheres are presented in the diagram. The analysis was performed with the DLT-Cam Viewer software and the measurements were made from two independent experiments with nine replications. (*—*p* < 0.05; ***—*p* < 0.0005).

In all tested lines, the size of the diameter of the spheroid was measured using the DLT-Cam Viewer software, statistical differences between the patient and the control course were determined at each hour of the test (Figure 5).

**Figure 6:**
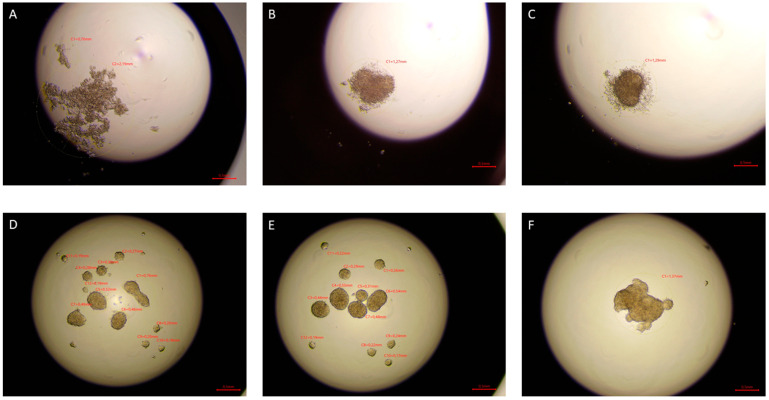
HEK293 cells in hanging drop culture. (**A**–**C**) show HEK293 cells from the control group 24, 48, and 72 h from the start of the culture, respectively. (**D**–**F**) show HEK293 cells exposed to 4.5 mT, 50 Hz EMF 24, 48, and 72 h from cultivation, respectively. Pictures taken with the Delta-Optical IB-100 microscope, 4× magnification.

**Figure 7:**
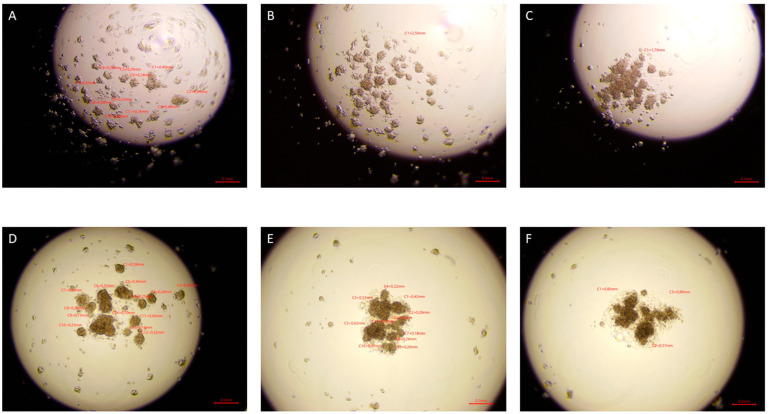
Caki1 cells in hanging drop culture. (**A**–**C**) show Caki1 cells from the control group 24, 48, and 72 h from the start of the culture, respectively. (**D**–**F**) show Caki1 cells exposed to 4.5 mT, 50 Hz EMF 24, 48, and 72 h from cultivation, respectively. Pictures taken with the Delta-Optical IB-100 microscope, 4× magnification.

**Figure 8:**
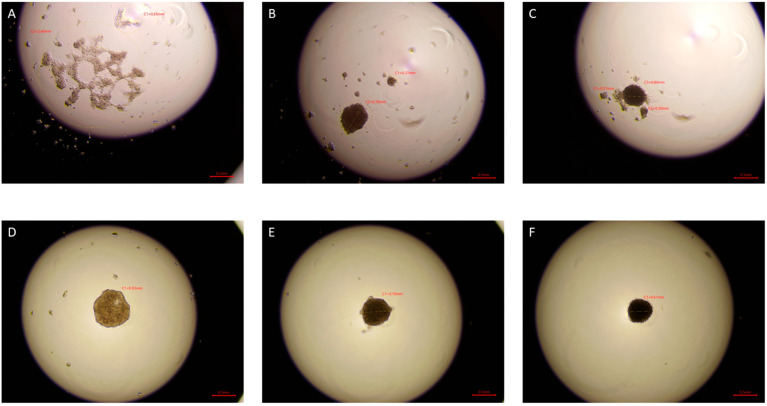
786-O cells in hanging drop culture. (**A**–**C**) show 786-O cells from the control group 24, 48, and 72 h from the start of the culture, respectively. (**D**–**F**) show 786-O cells exposed to 4.5 mT, 50 Hz EMF 24, 48, and 72 h from cultivation, respectively. Pictures taken with the Delta-Optical IB-100 microscope, 4× magnification.

**Figure 9:**
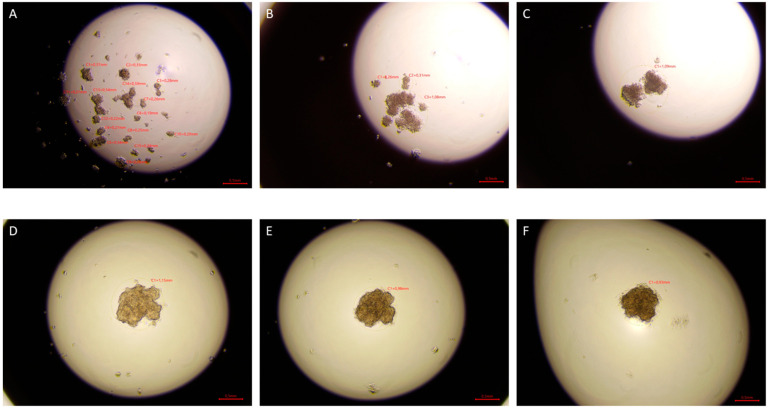
769-P cells in hanging drop culture. (**A**–**C**) show 769-P cells from the control group 24, 48, and 72 h from the start of the culture, respectively. (**D**–**F**) show 769-P cells exposed to 4.5 mT, 50 Hz EMF 24, 48, and 72 h from cultivation, respectively. Pictures taken with the Delta-Optical IB-100 microscope, 4× magnification.

After 72 h, tighter cell aggregates were formed in the drops of cells exposed to ELF-EMF in all tested lines, and a smaller number of spheres were formed compared to the control group. In the case of sham tumor lines (786-O, 769-P, and Caki1 not exposed to ELF-EMF), tight aggregates did not form even after 72 h of the study. The experiment was ended after 72 h. Figure 6, Figure 7, Figure 8 and Figure 9 show photos taken after 24, 48, and 72 h of culturing the tested cell lines from the control group and the test group in hanging drops.

## 3. Discussion

For many years, there has been debate among researchers regarding the role played by low-frequency electromagnetic fields on cancer cells. Various methods and research models have been used, but the results achieved are quite controversial and, unfortunately, divergent. Numerous studies have shown that exposure to ELF-EMF can also cause various changes at the cellular level in tumor cells. However, as mentioned at the beginning, there are no publications evaluating the effects of low-frequency EMF on clear cell renal carcinoma cells.

To determine the influence of the selected electromagnetic field on the research model, we decided to start by detecting possible morphological changes in the tested cell lines. In the conducted study, we didn’t observe any visible changes in the size or shape of the cancer cells tested. Rezaire-Taviraini et al., in their work, postulate that EMF with a frequency of 50 Hz affects the morphology of SH-SY5Y cells (neuroblastoma cell line), but without specifying any differences. In their work, researchers try to convince that the differences result from altered gene expression [10]. 

A more detailed study of the cytoskeleton of cells exposed to ELF-EMF would most likely show whether there were any significant changes in their morphology, although the differences should probably be sought in the extracellular matrix. In our study, the HEK293 line, which is considered to be the “healthy” kidney line, did not change its shape and size. The studies of Lai et al. also showed no morphological and histological changes in the kidney structure of rats exposed to ELF-EMF. Additionally, biochemical tests did not show any changes in kidney function of the tested animals [11].

The studied ELF-EMF parameters (4.5 mT, 50 Hz) resulted in the arrest of cancer cells in the G1 phase of the cell cycle (Figure 1), increasing the number of early apoptotic cells and decreasing the number of live cells in the 786-O lineage, as shown in Figure 2. Monache et al. in their research obtained contradictory results, explaining them with the so-called “biological window” of EMF effects, and especially with the hypothesis of “amplitude window” [12]. According to this theory, researchers suggest there are “specific allowed” levels that biosystems can reach under the influence of EMF. It is also related to the metabolic state of cells. Besides, the response of biological systems that are under different metabolic conditions may result in different results. Assuming that EMF affects only a small fraction of cells likely to be in a certain metabolic state or at a certain stage in their cell cycle, the duration of exposure to ELF-EMF can significantly change the recorded effects. Perhaps different results will be obtained when stimulating a larger population of cells. When the exposure conditions change, the electromagnetic field could also cause negative effects, increasing the probability of a thermal effect and, as a result, damaging tested cells.

The hanging drop test, as well as the migration and invasion assays, were used to assess the aggressiveness and metastatic capacity of the selected lines. Neoplastic ccRCC cells aggregated much faster when exposed to EMF than sham-exposed group, indicating that 4.5 mT, 50 Hz EMF might have an inhibitory effect on metastatic properties of ccRCC (Figure 7, Figure 8 and Figure 9). The HEK293 cell line aggregated the slowest from all tested lines also after exposure to EMF. As presented in Figure 6, there were no statistically significant differences between the size of aggregates. The reduced metastatic properties of tumor lines after EMF exposure are also confirmed by the results of the migration and invasion assays. This may be due to slight changes in the extracellular matrix, and not, as some scientists claim, the influence of EMF on the cytoskeleton of cancer cells [10,13,14].

When assessing tumor aggressiveness, it is also important to assess cell proliferation and viability. The resazurin based (TOX8) viability studies of the HEK293 line showed increased viability under the influence of EMF, in contrast to the cancer cells whose viability decreased. Similar results were obtained by Koziorowska et al., where they indicate that the most favorable exposure conditions are an electromagnetic wave with a sinusoidal shape and frequencies of 50 and 60 Hz [5]. The Provenzano et al. results also indicate that ELF-EMF promotes the differentiation of tretinoin-induced NB4 cells. After 96 h of exposure to 2 mT, cell numbers were approximately one-third lower compared to cells treated with tretinoin alone with no detectable signs of cell death [3]. The most favorable time to induce the death of neoplastic cells is a short exposure time, a maximum of 72 h [5,6,15,16].

It was initially proposed that ROS mediates the action of ELF-EMF on the biological system. Carefully coordinated regulation of intracellular ROS levels is an important process involved in the control of proliferation and differentiation in mammalian cells [17]. High levels of intracellular ROS are cytotoxic and lead to cell aging and death [18], while mild reduction of antioxidant-induced intracellular ROS levels increases cell proliferation by activating Akt and Erk1/2 pathways [19,20]. Interestingly, some studies suggest that exposure to ELF-EMF increases or decreases levels of intracellular ROS, thereby inducing various cellular effects. Exposure of 50 Hz ELF-EMF for 3 or 24 h has been reported to increase ROS production and DNA damage in rat fibroblasts [21]. On the other hand, exposure of the human microglial cell line to a 50 Hz EMF of 1 mT suppressed cell death by reducing intracellular ROS levels [22]. Exposure to 0.11 mT ELF-EMF increased the activity of the isoenzymes of superoxide dismutase and glutathione peroxidase, which are antioxidants to catalyze the reduction of intracellular ROS, thereby lowering intracellular ROS levels in murine squamous cells [23].

Song et al. observed elevated levels of p-Akt and p-Erk1/2, probably caused by exposure to uniform ELF-EMF, which may explain the increased cell proliferation rate induced by ELF-EMF [24]. Contrary to Song et al. shortly after termination of ELF-EMF exposure, we observed a significant increase in ROS levels, as did Provenzano et al. [3]. We also noticed increased ROS production in the HEK293 cell line (Figure 3), although it could have been since the cells were exposed to ELF-EMF outside the incubator. Examination of this in detail is quite important because the most sensitive to oxidative stress are the epithelial cells of the renal tubules and their damage may lead to kidney damage [25]. The mechanism behind ELF-EMF exposure to alter intracellular ROS levels should be further investigated to elucidate the biological effects of ELF-EMF.

Most of the scientists studying the effects of ELF-EMF claim they used a device to produce a “uniform ELF-EMF” while the device was placed in a CO_2_ incubator. The incubator, and especially its internal chambers, are usually made of metal, so the EMF to which the cells were exposed would not be uniform. A new ELF-EMF generator designed by Song et al. was expected to maintain the homogeneity of the EMF in the cell incubator. Their results showed that exposure to uniform ELF-EMF did not induce DNA damage or cell death, but rather promoted cell proliferation in both primary IMR-90 fibroblasts and HeLa cells in a time-dependent manner and magnetic field strength. The increased proliferation rate induced by ELF-EMF was reversible and did not modify cell cycle degradation [24]. Although in this study, the cells were kept for 30 min out of the incubator, the obtained results are similar to those obtained by Song et al., although in our study, the tumor cells showed reduced proliferation. Our results also indicate that 4.5 mT, 50 Hz ELF-EMF induced G0/G1 arrest, which could lead to apoptosis in cancer cells.

Our work shows a different effect of 4.5 mT, 50 Hz ELF-EMF exposure on ccRCC cancer cells and non-cancerous kidneys. These studies revealed that EMF could serve as a potential tool to manipulate cell viability through the involvement of ROS, possibly related signaling pathways, and genes important in characterizing cancer malignancy, opening up a new field of research valuable for future clinical applications. Despite the limitations of our study, we present important proof of concept showing that ELF-EMF can be considered a factor that inhibits the progression of ccRCC.

## 4. Materials and Methods

### 4.1. Cell Culture

The following adherent cell lines were purchased from American Type Culture Collection (LGC Standards, Łomianki, Poland): HEK293, 786-O, 769-P, and Caki1. All cell lines were cultured as monolayers in RPMI 1640 medium (Thermo Fisher Scientific, Warsaw, Poland) supplemented with 10% (*v*/*v*) fetal bovine serum (Thermo Fisher Scientific, Warsaw, Poland), 1% penicillin-streptomycin, and 1% L-glutamine (Thermo Fisher Scientific, Warsaw, Poland) in a humidified atmosphere of 95% and 5% CO_2_ and at 37 °C. 786-O and 769-P were one of the first ccRCC established cell lines. Both of them have characteristics of ccRCC and are most commonly used in ccRCC-focused research. Caki-1 is also a popular metastatic RCC cell line. Importantly, this cell line was proposed as a model system of proximal tubule epithelium, because of its ability to form a polarized layer that resembles well-differentiated kidney tissue [26].

### 4.2. ELF-EMF Exposure

All selected cell lines were studied during the exponential growth phase. The cultures were exposed to EMF or sham-exposed (the control cell cultures were not exposed to an ELF-EMF. However, all other conditions were identical to the experimental group). Exposure of cells to ELF-EMF did not introduce changes in the temperature of the plates or wells where the cells were located. Cells were seeded into a flat-bottom 6-well plate (25 × 10^3^ cells/well) and were cultured as monolayers. After 72 h of being plated, cells were continuously exposed to an electromagnetic field using a Helmholtz coil that generated a field at 50 Hz, and the magnetic induction was 4.5 mT. Parameters used in our study were selected based on the literature to observe changes while avoiding the thermal effect caused by EMF. Cells were exposed for 30 min a day for 5 days at room temperature. Cells from all plates on the fifth day, immediately after the end of the exposure, were detached using trypsin (Thermo Fisher Scientific, Warsaw, Poland). Then, the cells were counted with a T20 cell counter (Bio-Rad) supplemented with 10% trypan (Bio-Rad, Warsaw, Poland) and used for experiments.

### 4.3. Cells Viability

EMF and sham-exposed cells were seeded at a density of 2000 cells in 96-well plates and were cultured in RPMI-1640 medium with 10% fetal bovine serum and 1% penicillin-streptomycin and 1% L-glutamine (Thermo Fisher Scientific, Warsaw, Poland) at 37 °C with 5% CO_2_ and humidified atmosphere. After 72 h of incubation, the cell viability was analyzed using the resazurin test (In vitro TOX8, Sigma, Sigma, Aldrich, Poznań, Poland). 10% resazurin solution in complete RPMI 1640 medium was added to each well of the culture plate. Wells with culture medium containing no cells were used as blank. The prepared plates were incubated at 37 °C, 5% CO_2_ for 3 h. After this time, the absorbance was read at 570 nm and 600 nm in the FLUOstar Omega spectrophotometer (BMG Labtech, Ortenberg, Germany). The obtained results were analyzed according to the formula below:

$$\%\;viability=\;\frac{mean\;absorbance\;of\;cells\;exposed\;to\;ELF-EMF}{mean\;absorbance\;of\;cells\;from\;the\;sham\;group}\;\times 100\%$$

### 4.4. Hanging Drop Assay

From each cell line from both groups, a suspension of 2 × 10^6^ cells/mL was prepared. Then 10 µL of the cell suspension was placed on the lid of the Petri dish in nonuplicate. The lids with droplets were inverted and placed on a dish filled with PBS. The protocol described by Foty [27] was used for the hanging drop test. The dishes were incubated at 37 °C, 5% CO_2_ for 3 days. Pictures were taken after 24, 48, and 72 h of incubation using the Delta-Optical IB-100 microscope to compare the time of formation, morphology, and aggregate size (4× magnification).

### 4.5. Migration and Invasion Assay

To test the migratory and invasive abilities of the studied cell lines, a protocol prepared by Justus et al. [28] was used. For the migration and invasion assays, we used the insert with a pore diameter of 8 μm in a 24-well plate (HTS Multiwell Insert System 8.0 um pore size, 24 Multiwell format, Corning, Stryków, Poland). For the invasion test, a layer of 50 μL Matrigel (Coating Matrix Kit, Thermo Fisher Scientific, Warsaw, Poland) was previously added to the surface of the inserts and then incubated for 30 min at 37 °C and 5% CO_2_. In both, the migration and invasion assays, 100 μL of cell suspension was added to the surface of the insert and incubated for 10 min at 37 °C and 5% CO_2_ to allow cells to settle. After that, 600 µL of RPMI 1640, supplemented with 10% FBS and 1% penicillin and streptomycin, was added to the bottom of the lower chamber. The plates were incubated for 48 h at 37 °C and 5% CO_2_, after which the medium from the well and the insert was removed, and then the cells were fixed in 600 µL 70% ethanol for 10 min. Then the ethanol was removed, and the plates were allowed to dry. Then 600 µL of 0.2% crystal violet (Argenta, Poznań, Poland) was added to the bottom of the wells of the plates and the inserts were placed therein. The plates were incubated at room temperature for 10 min and then crystal violet was gently removed from the top of the membrane with a cotton applicator.

The membrane was carefully immersed in PBS solution (Thermo Fisher Scientific, Warsaw, Poland) 5 times to remove excess crystal violet but not to remove the cells. The bottoms of the insert wells were also washed gently five times with PBS solution. The plate was allowed to dry and the number of cells in different fields of view (mean from 5 fields at 40× total magnification) from the bottom of the plate wells and inserts was counted under a Delta Optical IB-100 microscope to obtain an average sum of cells that migrated through the membrane towards the chemoattractant and they attached themselves to the bottom side of the membrane.

### 4.6. Apoptosis and Cell Cycle Analysis

Both analyses were performed using FACSCalibur flow cytometer (Becton Dickinson, Warsaw, Poland).

Cell cycle analysis was performed using FxCycle PI/RNase kit (Thermo Fisher Scientific, Warsaw, Poland) and the analysis of apoptosis was performed by dual staining with Annexin V-FITC and propidium iodide (PI) (Thermo Fisher Scientific, Warsaw, Poland) following the manufacturer’s protocol. For both assays, 10^6^ cells/mL suspensions were used. Samples were analyzed using the Cell Quest and FCS Express 6 software.

### 4.7. Determination of ROS Generation

Fluorogenic CellROX Green Reagent probes (Thermo Fisher Scientific, Warsaw, Poland) were used to measure the oxidative stress in living cells. After exposure, CellROX^®®^ at a final concentration of 5 µM was added to all wells from both plates, and 3% H_2_O_2_ was added to three wells from the sham group. The cells were incubated for an additional 30 min at 37 °C, then collected by trypsinizing, washed three times with PBS solution, and finally resuspended in 300 µL PBS. Samples were analyzed using a FACSCalibur cytometer (Becton Dickinson, Warsaw, Poland) with the Cell Quest and FCS Express 6 software.

### 4.8. Statistical Analysis

Statistical evaluation of the obtained results was carried out using the two-way analysis of variance (ANOVA) for the results of cytometric tests, while the analysis of migration and invasion results was performed using the Student’s *t*-test. The evaluation of the size of the spheres in the hanging drop test showed an abnormal distribution. Therefore, the Mann–Whitney test was used to analyze the results. Results are presented as mean ± SD. The results at the level of *p* < 0.05 were considered statistically significant. The GraphPad Prism ver 7 Software (GraphPad Software, La Jolla, San Diego, CA, USA) and Microsoft Excel 2016 were used to perform statistical calculations. In the descriptions of results, the expression “relative to the control” means the sham group, which is a population of cells not exposed to ELF-EMF.

## Data Availability

The data presented in this study are openly available in Zenodo repository at http://doi.org/10.5281/zenodo.4477407.
